# Higher extrinsic and lower intrinsic connectivity in resting state networks for professional Baduk (Go) players

**DOI:** 10.1002/brb3.853

**Published:** 2017-10-30

**Authors:** William S. Sohn, Tae Young Lee, Seoyeon Kwak, Youngwoo Bryan Yoon, Jun Soo Kwon

**Affiliations:** ^1^ Institute of Human Behavioral Medicine SNU‐MRC Seoul Korea; ^2^ Department of Psychiatry Seoul National University College of Medicine Seoul Korea; ^3^ Department of Brain and Cognitive Sciences Seoul National University Seoul Korea

**Keywords:** Baduk, expertise, Go, resting functional magnetic resonance imaging, resting state

## Abstract

**Introduction:**

Dedication and training to a profession results in a certain level of expertise. This expertise, like any other skill obtained in our lifetime, is encoded in the brain and may be reflected in our brain's connectome. This property can be observed by mapping resting state connectivity. In this study, we examine the differences in resting state functional connectivity in four major networks between professional “Baduk” (Go) players and normal subjects.

**Methods:**

Resting state fMRI scans were acquired for professional “Baduk” (Go) players and normal controls. Major resting state networks were identified using independent component analysis and compared between the two groups. Networks which were compared include the default mode network, the left and right fronto‐parietal network, and the salience network.

**Results:**

We found that normal subjects showed increased connectivity within certain areas of each target network. Professional players, however, showed higher connectivity to regions outside the traditional regions of each given network. Close examination of these regions revealed that regions shown to have higher connectivity in professional players have been revealed to be relevant in expertise for board games.

**Conclusion:**

The findings in this study suggest that continuous training results in greater integration between regions and networks, which are necessary for high‐level performance. The differences observed in our study between normal controls and professional players also shed light on the difference in brain connectivity which can arise through lifestyle and specialization in a specific field.

## 1 INTRODUCTION

The use of functional imaging has allowed insight into the overall functional organization of the brain and its uniqueness across individuals. Current functional magnetic resonance imaging (fMRI) studies have shown that the brain's connectivity plays a large part in cognitive ability (Bullmore & Sporns, 2012). This functional “connectome” is believed to be shaped by the learning and experiences that are accumulated over an individual's lifetime (Finn et al., 2015; Wang et al., 2015). This is described in Hebbian theory, which hypothesizes that neurons that co‐activate during a task develop connections among one another (Brown & Milner, 2003; Favero, Cangiano, & Busetto, 2014). Therefore, repeated experiences or training will result in the formation of connections or networks in the brain that are specialized for a certain task or can be used to facilitate similar tasks (Chavan, Mouthon, Draganski, van der Zwaag, & Spierer, 2015; Debowska et al., 2016; Fink et al., 2015; Sun et al., 2016; Zhang, Yao, Shen, Yang, & Zhao, 2015).

Expertise indicates expert skill or knowledge in a given field of study or profession. Obtaining such a level of proficiency requires a large amount of time, often a lifetime, dedicated to a specific field. These experiences and specializations are thought to lead to more efficient organizations of task‐related networks in the brain. Studies have shown unique activation or recruitment of cognitive resources in professionals in a variety of fields (Bangert et al., 2006; Bernardi et al., 2013; Hou, Chen, & Dong, 2015; Ito, Matsuda, & Shimojo, 2015; Kashkouli Nejad et al., 2014; Lu et al., 2015; Meister et al., 2004; Spada, Verga, Iadanza, Tettamanti, & Perani, 2014). Board game experts provide a unique opportunity to study people who have obtained a high level of competence in their field through extensive training. Many studies have examined the differences in functional connectivity in professional board game players (Duan et al., 2012; Jung et al., 2013; Li et al., 2015). These studies show that highly trained professionals are different not only their increased ability for tasks related to their field of expertise but also in the way their brain responds to solve these tasks.

The game of “Baduk,” more commonly known as “Go,” is a complex game requiring in‐depth mental strategies. Despite its simple rules, the game is very complex and requires high levels of visuospatial attention, working memory, and decision‐making to perform at an expert level. The complexity of the game is illustrated best by the number of possible outcomes that may occur. This number is so large that computer algorithms have only recently begun to consistently outperform professional players (Silver et al., 2016). This feat requires massive computational power along with deep‐learning algorithms. Therefore, as with any other field, a lifetime of dedication to this game is often still not sufficient for complete mastery. Current studies have revealed differences in the network properties of professional players versus normal controls, suggesting greater efficiency of cognition in players (Chen et al., 2003; Duan et al., 2012; Jung et al., 2013). In addition to functional differences, white matter studies have also revealed structural differences and stronger connectivity in regions associated with attentional control, working memory, and problem‐solving when trained in the game of “Go” (Lee et al., 2010). These results suggest that the neurocognitive load required to play “Go” may have therapeutic and advantageous effects for development (Kim et al., 2014; Newman, Hansen, & Gutierrez, 2016).

In this study, we examine the differences in resting state connectivity for four major resting state networks between professional “Baduk” players and normal subjects. We hypothesize that the differences in cognitive abilities of each subject group can be reflected in the resting connectivity in major resting state networks. In addition, we hypothesize that professional players will show higher resting connectivity to regions of the brain which are commonly activated in previous literature during a board game task.

## 2 MATERIALS AND METHODS

### 2.1 Subject demographics

A total of 44 subjects were recruited for this experiment. Participants included 23 professional players along with 20 controls (Table 1). Differences in age, education, and IQ were compared using a two‐tailed *t test* and differences in gender were compared using a chi‐squared test. One control subject was excluded from this dataset due to poor fMRI data quality.

**Table 1:** Demographics showing age, sex, education, and IQ of Professional players (*N* = 23) and normal controls (*N* = 20)

	NL	PRO	*p*‐value
*N*	20	23	N/A
Age	21.1 ± 1.9	22.8 ± 3.3	.05
Sex	13 M:7 F	17 M:6 F	.53
Edu	14.0 ± 1.3	12.4 ± 2.8	.02
IQ	110.2 ± 11.4	107 ± 14.3	.54

### 2.2 fMRI acquisition parameters

The blood oxygenation level‐dependent (BOLD) signal was acquired using a 3T Siemens Trio MRI scanner (Siemens Healthcare, Erlangen, Germany) using T2‐weighted gradient echo planar imaging (repetition time [TR]=2000 ms; echo time [TE]=30 ms; field of view [FOV]=220 mm; flip angle=90°; 4 mm thickness; 27 axial slices; matrix= 64 × 64). T1 anatomical volume reference images were also acquired (TR=1670 ms; TE=1.89 ms; FOV=250 mm; flip angle=9°; 1 mm thickness; 208 slices; matrix=256 × 256). Foam pads were used to reduce head motion.

### 2.3 MRI preprocessing

Resting and functional fMRI data were subjected to the same preprocessing pipeline using FSL (FMRIB Software Library, http://fsl.fmrib.ox.ac.uk/fsl/fslwiki/). Skull‐stripping fMRI and structural MRI were performed using BET (Brain Extraction Toolbox). Image registration and normalization was performed using FLIRT (FMRIB's Linear Registration Tool) with an affine volume registration. Structural MRI results were segmented using FAST (FMRIB's Automated Segmentation Tool). The resulting segmented images for white matter and CSF were thresholded (<0.7), binarized, and normalized to MNI space using the transformation matrix obtained in FLIRT. Time‐series were extracted for white matter and CSF masks and were regressed out of fMRI data using GLM. fMRI results then underwent slice timing correction, temporal high‐pass filtering (Gaussian‐weighted least‐squares line fitted with sigma=100.0 s), MCFLIRT motion correction, and spatial smoothing (Gaussian kernel of FWHM 4 mm). Smoothing was set to 4 mm to reduce loss in spatial resolution in the hopes of identifying small subcortical structures. Subjects were also checked for excessive head motion by calculating the average framewise displacement (FD). The first two scans were discarded.

### 2.4 Resting state analysis

Group independent component analysis (ICA) was performed using the MELODIC (Multivariate Exploratory Linear Optimized Decomposition into Independent Components) toolbox in FSL. The output component number was set to 25 components. Component output number was selected based on recommended values for obtaining consistent networks with whole brain ICA (Abou Elseoud et al., 2011; Damoiseaux et al., 2006; Ray et al., 2013). Four common major resting state networks were identified from the output network for analysis. These networks include the default mode network (DMN), the left and right fronto‐parietal networks (FPNL, FPNR), and the salience network (SAL). Networks were then reconstructed for each individual directly from ICA time‐series as outlined in a previous study (Sohn et al., 2015). Group analyses were performed using the higher level analysis in the FEAT toolbox and compared with an unpaired *t* test using a cluster correction of <0.05.

## 3 RESULTS

Statistical analysis of demographics revealed a significant difference in education and an uncorrected *p*‐value of .05 in age (Table 1). Average age for normal subjects was 21.1 ± 1.9 years and for professionals, was 22.8 ± 3.3 years. Average education years for normal subjects was 14.0 ± 1.3 years and for professionals, was 12.4 ± 2.8 years.

**Figure 1:**
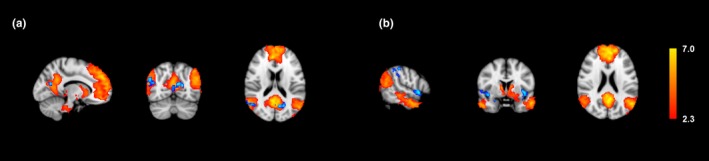
Average default mode network connectivity and differences between professional players and normal controls. Group averages are obtained for normal controls and professional players and compared for differences. Group averages and increased resting connectivity are shown for normal controls (a) and professional players (b). Higher connectivity for each group is shown in blue, while average resting correlation maps for each group is shown in red/yellow

**Figure 2:**
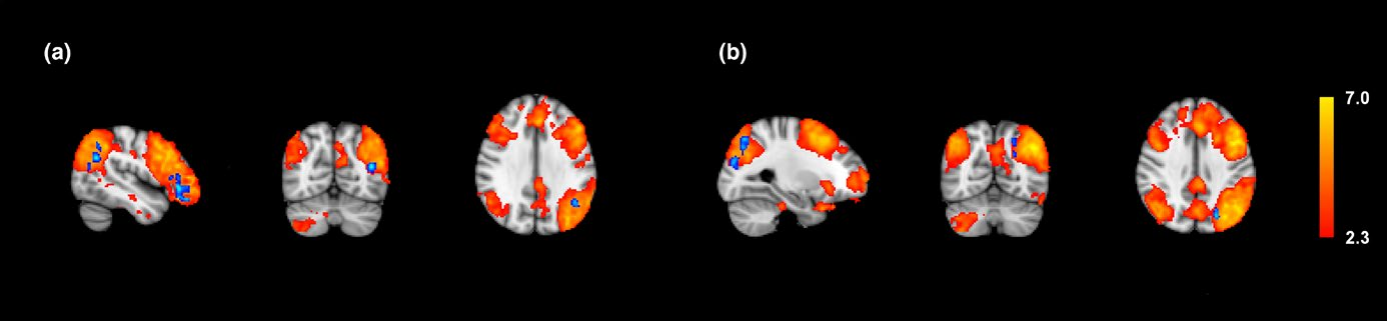
Average left fronto‐parietal network connectivity and differences between professional players and normal controls. Group averages are obtained for normal controls and professional players and compared for differences. Group averages and increased resting connectivity are shown for normal controls (a) and professional players (b). Higher connectivity for each group is shown in blue, while average resting correlation maps for each group is shown in red/yellow

**Figure 3:**
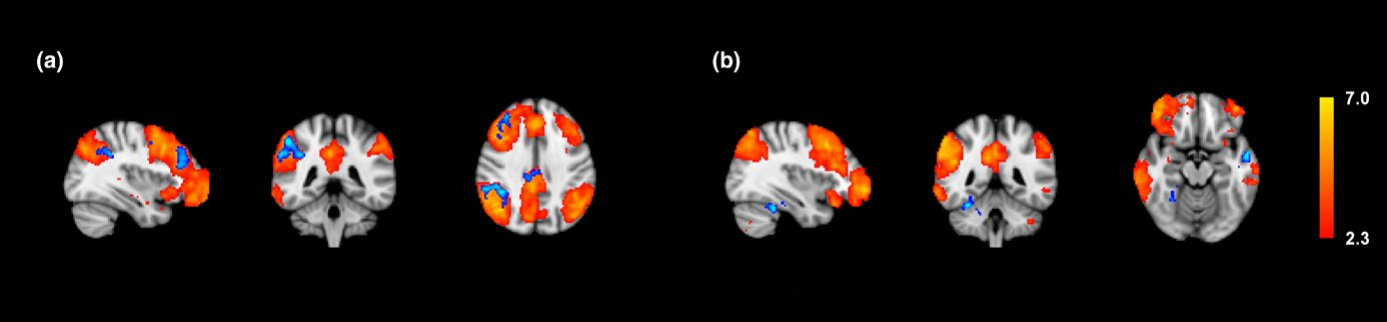
Average right fronto‐parietal network connectivity and differences between professional players and normal controls. Group averages are obtained for normal controls and professional players and compared for differences. Group averages and increased resting connectivity are shown for normal controls (a) and professional players (b). Higher connectivity for each group is shown in blue, while average resting correlation maps for each group is shown in red/yellow

**Figure 4:**
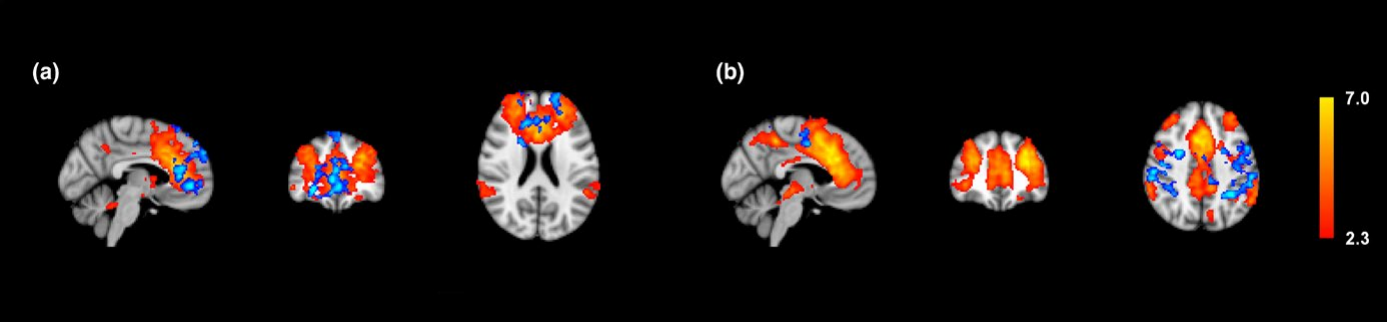
Average salience network connectivity and differences between professional players and normal controls. Group averages are obtained for normal controls and professional players and compared for differences. Group averages and increased resting connectivity are shown for normal controls (a) and professional players (b). Higher connectivity for each group is shown in blue, while average resting correlation maps for each group is shown in red/yellow

Four major resting state networks were compared for differences in resting state connectivity. The average connectivity maps for each group were obtained, and differences were mapped for each network (Figures 1, 2, 3, 4ab). Overall, normal subjects displayed activity in regions traditionally associated with each network. In the DMN, higher connectivity was observed in normal subjects within the precuneus and the right lateral parietal lobe (Figure 1a). In the FPNL, normal subjects showed higher resting connectivity in the left frontal, lateral occipital, and parietal lobes (Figure 2a). In the FPNR, higher resting connectivity was observed in the right frontal lobe (Figure 3a). Finally, the SAL network showed higher connectivity in the anterior cingulate cortex (ACC), medial frontal cortex (MPFC), and superior frontal lobe (Figure 4a).

Compared to normal subjects, the professional players generally showed greater extrinsic connectivity or connectivity outside traditional regions of each network. In the DMN, professional subjects showed greater resting connectivity to the insular cortex, supramarginal gyrus, and temporal pole (Figure 1b). In the FPNL, greater connectivity was shown in the left superior parietal lobe (Figure 2b). In the FPNR network, greater connectivity was observed in the middle and superior temporal gyrus and the fusiform gyrus (Figure 3b). In the SAL network, greater connectivity was observed in the sensorimotor cortex and the supramarginal gyrus (Figure 4b).

## 4 DISCUSSION

Resting state research has shown robust connectivity in major resting state networks across subject groups and studies (Buckner, Krienen, & Yeo, 2013; Damoiseaux et al., 2006; De Domenico, Sasai, & Arenas, 2016). The reproducibility and relative ease of image acquisition has made these resting state networks an ideal focus of neuroimaging studies. Therefore, many studies implement resting state designs to study neuroplasticity as it pertains to training and development (Klados, Styliadis, Frantzidis, Paraskevopoulos, & Bamidis, 2016; Klein, Liem, Hanggi, Elmer, & Jancke, 2016; Smit, de Geus, Boersma, Boomsma, & Stam, 2016; Sun et al., 2016; Yoo, Sohn, & Jeong, 2013). In our study, we propose that lifelong training in the game of “Go” results in increased resting state connectivity to regions of the brain that are necessary for high‐level performance in board game playing.

Four of the most prominent networks were investigated in this study. These networks include the DMN, FPNL, FPNR, and SAL. Overall, control subjects had higher intrinsic connectivity within each network than professional players. One exception was the FPNL network, which showed increased connectivity to areas inside the left parietal lobe (Figure 2b). Higher intrinsic connectivity in resting state network for normal controls can possibly be attributed to lifestyle. In general, professional players in this study began playing at an early age. As a result, their daily routines and lifestyles were and are different than nonplayers. The decreases in intrinsic network connectivity revealed by our analysis may be reflective of the differences in daily lifestyles between professional players and normal controls. Studies have shown that the cultural background can affect functional connectivity (Goh et al., 2013; Hedden, Ketay, Aron, Markus, & Gabrieli, 2008; Krendl, 2016). Therefore, decreases in intrinsic connectivity may be reflective of professional players’ lack of a need for the high correlation of these regions in their lifestyles. In addition, different training and experiences may have resulted in a means to process information. This result can be exemplified by the increases in extrinsic connectivity observed in professional players. Further studies are needed to confirm the effect of lifestyle on intrinsic network connectivity.

Professional players show increases in functional connectivity for each network in areas that are traditionally not part of these networks. While functions and tasks can largely be managed by one specific task network, research has begun to reveal that function is associated with the dynamic interplay between brain regions (Finn et al., 2015; Zuo et al., 2012). Therefore, increased connectivity to these regions may represent different ways that professional players mentally process certain tasks.

In our study, we demonstrated increased connectivity of the DMN to the insular cortex in professional players. The insula has many functions, including consciousness, emotional awareness, and decision‐making (Gu, Hof, Friston, & Fan, 2013; Ishii, Ohara, Tobler, Tsutsui, & Iijima, 2012). One study found that patients with lesions in the insular cortex failed to properly adjust for the odds of winning. In fact, inactivation of the anterior insular cortex has been linked to a propensity to take fewer risks, suggesting that the increased connectivity to the insula can possibly represent a higher propensity of professional players to take risks, a trait that may be necessary to be successful in their field (Clark et al., 2008; Ishii et al., 2012). Other studies have shown that the right insula crucial for regulating decision tasks plays a casual role in the DMN (Chand & Dhamala, 2016a,b; Sridharan, Levitin, & Menon, 2008). Thus, this increase in connectivity of the DMN to the insular cortex may be a result of increased calculated risk‐taking, which may have developed from consistent board game playing.

One network that is more closely related to cognitive ability and board game playing is the FPNR. The FPN is largely associated with cognitive control and attention (Cole, Repovs, & Anticevic, 2014; Scolari, Seidl‐Rathkopf, & Kastner, 2015; Zanto & Gazzaley, 2013). Studies have suggested that the FPN plays a major role in visual attention, particularly space‐based attention (Yantis & Serences, 2003). This function can be related to playing “Go,” as the opponents’ pieces can appear in expected and unexpected areas. Our study found increased connectivity of the FPNR to the right FFA and left MTL. Studies of chess players have shown that differences in skill are largely associated with “chunks” held in long‐term memory (Amidzic, Riehle, Fehr, Wienbruch, & Elbert, 2001). The MTL plays a major role both in memory encoding and retrieval (Song, Jeneson, & Squire, 2011; Squire, Wixted, & Clark, 2007; Squire & Zola‐Morgan, 1991). The increased connectivity between the FPNR and the MTL may be reflective of greater integration between memory retrieval and the neural processes of visuospatial attention and perception. The FFA region is related to this process. While the FFA is normally associated with face recognition, studies have shown that expert chess players show higher activation than novice players in this region when presented with chess stimuli (Bilalic, Langner, Ulrich, & Grodd, 2011; Boggan, Bartlett, & Krawczyk, 2012). These studies suggest that the FFA region plays a larger role than facial recognition and possibly encompasses expert level object recognition as well (Boggan & Huang, 2011). Increased connectivity to the FFA region in professional players suggests a greater integration among regions responsible for cognitive control, visuospatial attention (FPN), and expertise in object/pattern recognition.

Finally, the SAL network is responsible for a variety of tasks, including initiating cognitive control, performing and maintaining tasks, and coordination (Dosenbach et al., 2006; Medford & Critchley, 2010; Seeley et al., 2007). During task maintenance, regions of the SAL network, particularly the ACC, have been shown to play a role in error processing (Debener et al., 2005; Ham, Leff, de Boissezon, Joffe, & Sharp, 2013; Holroyd et al., 2004). Previous studies have shown activation in regions of the motor cortex while performing “Go”‐related tasks (Chen et al., 2003). The increase in connectivity of the salience network with other brain regions can act to modulate actual motor activity while playing to limit errors.

One thing to note is that there were significant differences in the number of education years. This is largely because professional “Go” players usually forgo traditional education in favor of more focused training in their profession. As a result, professional players attend traditional educational institutions shorter than normal subjects. In addition, there was a *p*‐value of .05 in age differences. However, since the age gap between the two groups is so low (1.7 years), it is likely that this will not affect our results as functional connectivity does not change much in adolescence/early adulthood (Chan, Park, Savalia, Petersen, & Wig, 2014).

Professional sports are unique in that players typically begin their profession at a very young age, which is different from other professions that are often learned after college. This can lead to a vast difference in how an individual trains and prepares for their future. The expertise obtained over years of board game playing reveals distinct differences in functional connectivity in four major resting state networks compared to normal subjects. In general, we found that normal subjects showed increased connectivity within certain areas of each target network, while professional players showed higher connectivity to regions outside traditional areas of each given network. These regions have often been shown to be important in board game playing, suggesting that the network differences observed in our study are a result of continuous training to facilitate and improve the performance of key skills needed to excel in professional board game playing.
